# Supplementary material for: Effectiveness of the Ticket to Work program in supporting employment among adults with disabilities
Source: medRxiv. 2025 Apr 22:2025.04.22.25325884. Preprint. [Version 1] doi: 10.1101/2025.04.22.25325884 (PMC12045388; doi:10.1101/2025.04.22.25325884)
Supplement: Supplement 1 [file NIHPP2025.04.22.25325884v1-supplement-1.pdf]

medRxiv preprint doi: https://doi.org/10.1101/2025.04.22.25325884; this version posted April 22, 2025. The copyright holder for this preprint (which was not certified by peer review) is the author/funder, who has granted medRxiv a license to display the preprint in perpetuity. This article is a US Government work. It is not subject to copyright under 17 USC 105 and is also made available for use under a CC0 license.

Appendices A. Supplementary Figures

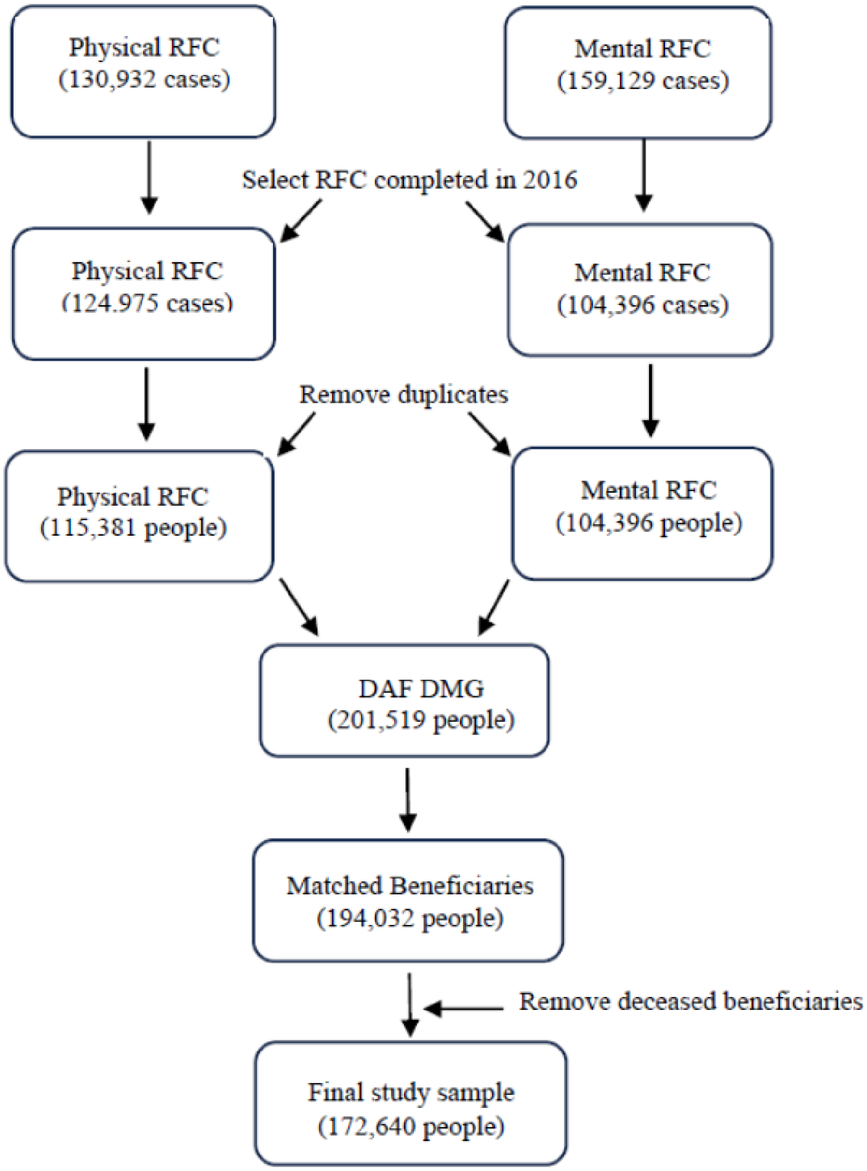

Supplemental Fig. A1. Sample Flowchart

medRxiv preprint doi: https://doi.org/10.1101/2025.04.22.25325884; this version posted April 22, 2025. The copyright holder for this preprint (which was not certified by peer review) is the author/funder, who has granted medRxiv a license to display the preprint in perpetuity.
This article is a US Government work. It is not subject to copyright under 17 USC 105 and is also made available for use under a CC0 license.

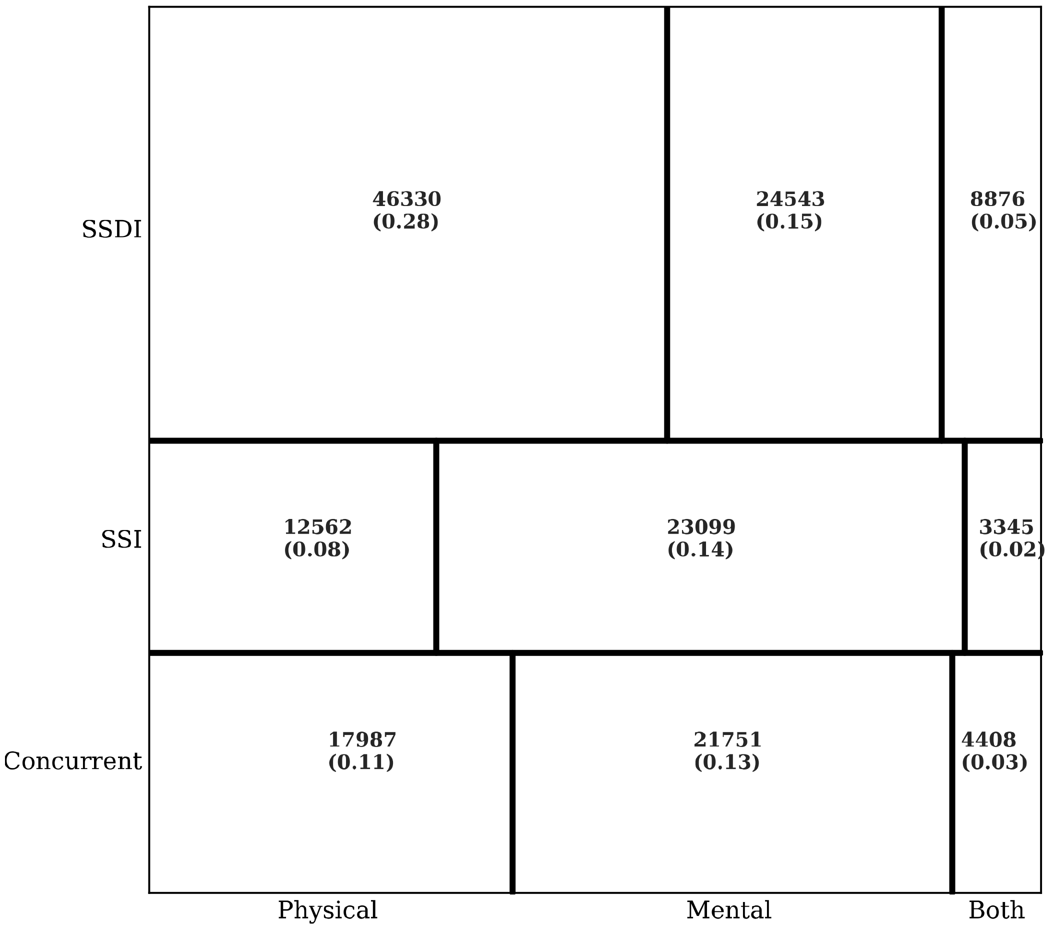

Supplemental Fig. 2. Contingency table used in parameter scaling for the Bayesian hierarchical model

medRxiv preprint doi: https://doi.org/10.1101/2025.04.22.25325884; this version posted April 22, 2025. The copyright holder for this preprint (which was not certified by peer review) is the author/funder, who has granted medRxiv a license to display the preprint in perpetuity. This article is a US Government work. It is not subject to copyright under 17 USC 105 and is also made available for use under a CC0 license.

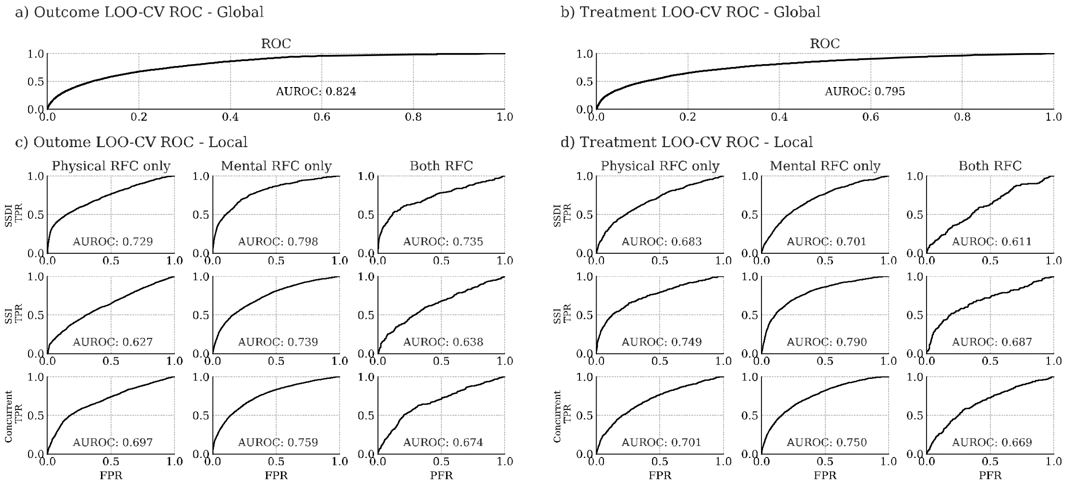

Supplemental Fig. 3. Global and local within-cohort-wise leave one out cross-validated (LOO-CV) area under the receiver (AUROC) curves for assessing the generalizability accuracy of the Bayesian joint outcome hierarchical model on both outcome and treatment assignment prediction.
